# Birth outcomes and early growth patterns associated with age at adiposity rebound: the Ma’anshan birth cohort (MABC) study

**DOI:** 10.1186/s12889-023-17236-9

**Published:** 2023-12-04

**Authors:** Jixing Zhou, Yuzhu Teng, Shanshan Zhang, Mengting Yang, Shuangqin Yan, Fangbiao Tao, Kun Huang

**Affiliations:** 1 Department of Maternal, Child and Adolescent Health, School of Public Health, Anhui Medical University, Hefei, 230032 China; 2 Key Laboratory of Population Health Across Life Cycle (AHMU), MOE, Hefei, 230032 China; 3 NHC Key Laboratory of Study on Abnormal Gametes and Reproductive Tract, Hefei, 230032 China; 4 Anhui Provincial Key Laboratory of Population Health and Aristogenics, Hefei, 230032 China; 5 Maternal and Child Health Care Center of Ma’anshan, No 24 Jiashan Road, Ma’anshan, Anhui 243011 China; 6 Scientific Research Center in Preventive Medicine, School of Public Health, Anhui Medical University, Anhui Province, China

**Keywords:** Adiposity rebound, Birth outcome, Rapid weight gain, BMI trajectory, Children

## Abstract

**Objective:**

Early onset of adiposity rebound (AR) is considered an early indicator of obesity risk. Our objective was to investigate the association of birth outcomes and early physical growth patterns with early AR in children.

**Methods:**

Study subjects (n = 2705) were enrolled from the Ma’anshan birth cohort (MABC). The body mass index (BMI), head circumference, waist circumference, and body fat were collected. Rapid weight gain (RWG) was defined by the change in weight standard-deviation score in the first two years of life. Group-based trajectory modeling (GBTM) was used to determine children’s physical growth trajectories. The age of AR was fitted using fractional polynomial function models.

**Results:**

Children with very high BMI trajectories (RR = 2.83; 95% CI 2.33 to 1.40), rising BMI trajectories (RR = 3.15; 95% CI 2.66 to 3.72), high waist circumference trajectories (RR = 4.17; 95% CI 3.43 to 5.06), and high body fat trajectories (RR = 3.01; 95% CI 2.62 to 3.46) before 72 months of age were at a greater risk of experiencing early AR. Low birth weight (LBW) (RR = 1.86; 95% CI 1.28 to 2.51), preterm birth (PTB) (RR = 1.50; 95% CI 1.17 to 1.93), and small for gestational age (SGA) (RR = 1.37; 95% CI 1.14 to 1.64) associated with increased risk of early AR. Moreover, infants experiencing RWG (RR = 1.59; 95% CI 1.40 to 1.83), low BMI trajectories (RR = 1.27; 95% CI 1.06 to 1.53) and rising BMI trajectories (RR = 1.50; 95% CI 1.22 to 1.84) in the first two years were at higher risk of developing early AR subsequently. Compared to the group with non-early AR, the BMI of children with early AR tended to be lower first (from birth to 6 months of age) and then higher (from 18 to 72 months of age).

**Conclusions:**

Children with overall high BMI, high waist circumference, and high body fat before 72 months of age are more likely to experience early AR, but infants with low BMI trajectories, rising BMI trajectories and infants experiencing RWG in the first two years of life similarly increase the risk of early AR. These results can help to understand the early factors and processes that lead to metabolic risks.

**Supplementary Information:**

The online version contains supplementary material available at 10.1186/s12889-023-17236-9.

## Introduction

The body mass index (BMI) pattern in humans during childhood presents an interesting phenomenon. The first peak value occurs around the first year after birth, followed by a period of decline until the onset of adiposity rebound (AR), and then the BMI slowly increases again [1]. AR was first reported in 1984 by Rolland-Cachera et al. [1], who identified the timing of AR as an early physiological predictor of obesity in late childhood and adulthood. Studies have confirmed that early age at AR is associated with an increased risk of diabetes [2], cardiometabolic disease [3], and other obesity-related comorbidities [4]. A recent systematic review reported that the overall prevalence of early AR (age at AR < 5.0-5.1 years) is 40%, and it is continuously increasing, with a particularly high overall prevalence of 52% in children born in 2002–2009 [5].

AR is an important physiological milestone during the early stages of physical growth. Exploring AR with changes in physical development in children will lead to an understanding of the early factors and processes that contribute to metabolic risk. Despite the widespread interest in the long-term health effects of age at AR, there is limited understanding of the causes of earlier age at AR. Bhargava et al. [6] found that children with the earlier AR had a higher BMI in late childhood and persisted into adulthood while having the lowest BMI at birth and at 2 years of age. Furthermore, they found a slight increase in BMI from birth to two years of age in individuals [6]. Rolland and Cole [7] also reported that the BMI trajectory of individuals with early age at adiposity rebound (EAR) was characterized by average or low levels of BMI before or after rebound and a rapid increase in gain in BMI after rebound. However, Koyama et al. [8] revealed no association between body weight gain during infancy and the timing of AR. Furthermore, there is inconsistency in the understanding of adverse birth outcomes in relation to the onser of AR. Results from limited cohort studies suggest an earlier age of AR in children with small for gestational age (SGA) compared with non-SGA [9, 10]. It has also been proposed that the higher the birth weight, the earlier the age of AR onset [11]; children with large for gestational age (LGA) are more likely to develop early AR than those with appropriate for gestational age (AGA) [12]. Eriksson et al. [13] observed that early AR was not associated with BMI, birthweight at birth.

There is not enough clarity about the association between early childhood physical growth patterns and early AR. High-density repeated measures of physical growth based on infancy and preschool years may help to further deepen the comparison of early physical growth patterns in children with different AR ages. In addition. existing studies have mostly focused on BMI, and it is not clear whether other physical indicators of the human body, such as head circumference, body fat and body weight, are similarly associated with AR.

Therefore, the present study aimed to investigate the relationship of birth outcomes and early growth patterns with age at AR based on a cohort study in China.

## Methods

### Study population

**Fig. 1:**
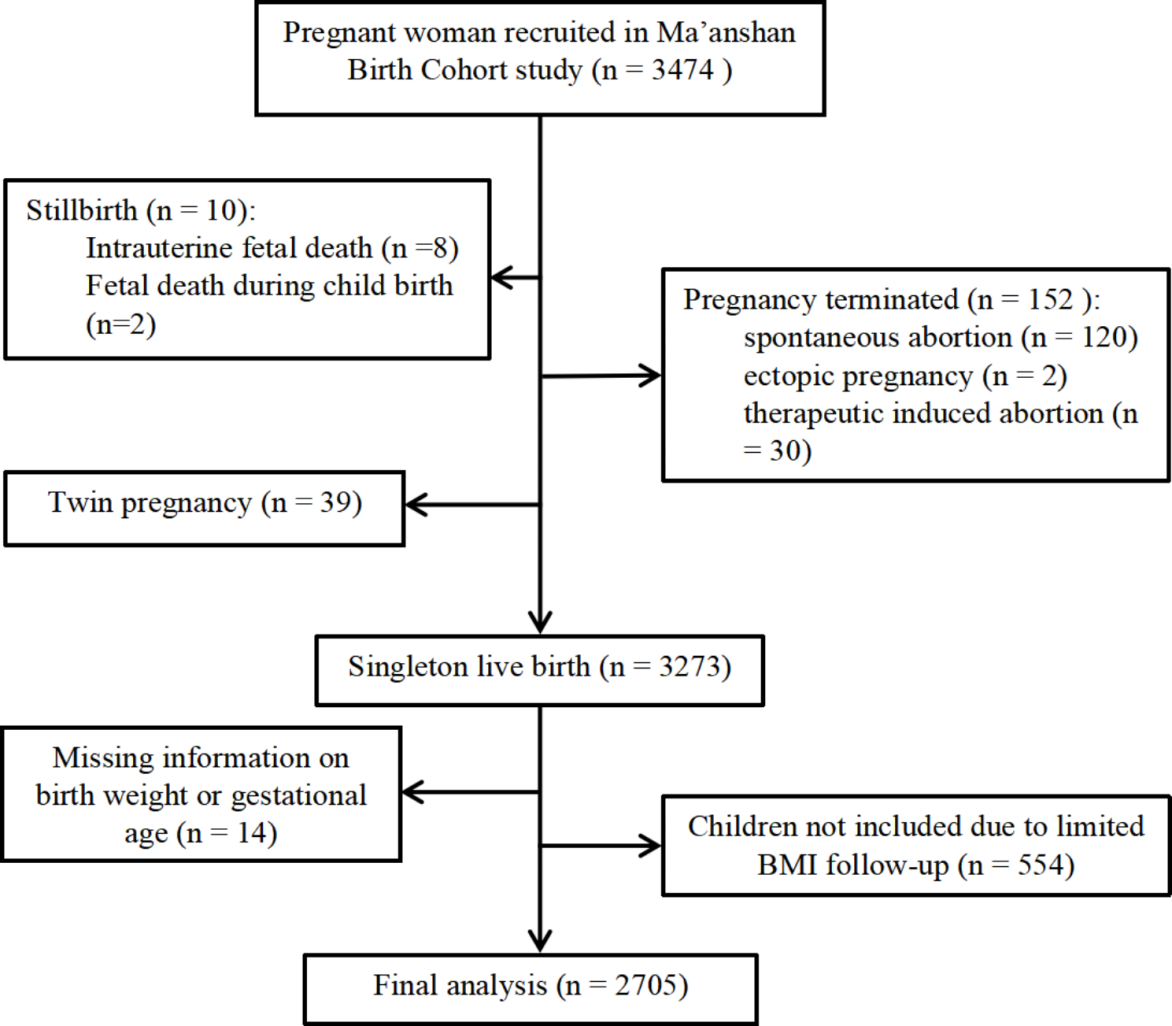
The follow-up diagram of the study participants

Participants (n = 3474) in this study were from the Ma’anshan Birth Cohort Study (MABC), an ongoing longitudinal prospective birth cohort study in Ma’anshan City, Anhui Province, China. Women (age > 18 years and gestational age < 14 weeks) who planned to undergo prenatal and childbirth follow-up visits at the Maternal and Child Health Center in Ma’anshan City were recruited from May 2013 to September 2014. The detailed inclusion criteria were described in our previous study [14]. Additional exclusion criteria for this study were as follows: (1) adverse pregnancy outcomes (embryo arrest, spontaneous abortion, therapeutic abortion, stillbirth, and ectopic pregnancy) (n = 162) and multiple pregnancies (n = 39); (2) children with missing information on birth weight or gestational age (n = 14); (3) children whose BMI was recorded less than six times from birth to six years of age (n = 554) (that is less than twice from 0 to 9 months; less than twice from 12 to 36 months and less than twice from 42 to 72 months). Finally, 2705 pairs of mother–child were included in the study. The recruitment of participants is shown in Fig. 1.

The study procedure was approved by the Ethics and Research Committee of Anhui Medical University (No. 20,131,195). All participants provided their informed consent in writing.

### Birth outcomes

The characteristics of the newborn, including the sex of the infant, gestational age, birth length and birth weight, were obtained from medical records. Based on gestational age and birth weight, infants were classified into preterm birth (PTB), non-PTB, SGA, AGA, LGA, low birth weight (LBW), adequate birth weight (ABW) and high birth weight (HBW). PTB and non-PTB were defined as the birth of infants ≤ 37 weeks and > 37 weeks of gestation, respectively [15]. SGA, AGA, and LGA were defined as infants whose birth weight was below the 10th percentile, between the 10th -90th percentile, and above the 90th percentile of infants of the same gestational age and sex, respectively [16]. LBW, ABW, and HBW were defined as newborn birth weights < 2500 g, 2500–3999 g, and ≥ 4000 g at birth, respectively [17].

#### Early physical growth

The physical growth of the children was repeatedly evaluated from birth to 6 years of age. Five follow-up visits were made in the first year, i.e., at 42 days, 3 months, 6 months, 9 months, and 12 months, after delivery. Subsequently, the children underwent anthropometric measurements every six months until 6 years of age. Anthropometric measurements were performed by pediatricians at Ma’anshan Maternal and Child Health Hospital.

The height and weight were measured at 16 follow-up visits from birth to 72 months. The body weight of the infants was measured with a pan-type lever scale of 0.01 kg; and the weight of the toddlers was measured with a seated lever scale of 0.05 kg. The length of the infant was evaluated with a standard measuring bed, accurate to 0.1 cm; and height and weight in children aged 3 to 6 years were measured with a mechanical height and weight scale (model: RRZ-50-RP), accurate to 0.1 kg for weight and 0.1 cm for height, respectively. During the measurements, children were asked to remove their shoes and hats and wear light clothing. The BMI was calculated using the following formula: BMI = weight (kg)/height (m^2^).

The children’s head circumference was measured at birth and at 42 days, then at 3, 6, 9, 12, 18, and 24 months after delivery. It was measured by trained pediatricians using a non-stretchable tape around the maximum diameter of the forehead and occiput, accurate to 0.1 cm. Waist circumference was measured at 30, 36, 42, 48, 54, 60, 66, and 72 months of age. The children were asked to remain standing, with the abdomen relaxed, and a soft measuring tape was placed 1 cm above the umbilicus of the child, with an accuracy of 0.1 cm. Body fat was evaluated at 48, 54, 60, 66, and 72 months of age and was measured using an InBody J20 body composition analyzer. The test requires the child to cooperate and remain stationary for 1–2 min to obtain the body fat content.

Weight-for-age z-score (WAZ), BMI-for-age z-score (BMIZ), and head circumference -for-age z-score were determined and classified using the World Health Organization (WHO) child growth standards and growth reference data [18].

### Early growth patterns

#### Rapid weight gain

Weight gain of the children was determined by the difference in WAZ between birth and 12, 18, and 24 months (∆z-scores = z-scores _larger months_ - z-scores _smaller months)_. We defined the criteria for RWG in childhood as a change in the SD score > + 0.67 from birth to 12, 18, and 24 months, which is the most frequent and widely accepted definition of RWG [19]. Early childhood weight gain was classified as RWG (∆z-scores > 0.67) and non-RWG (∆z-scores ≤ 0.67).

#### Estimation of the trajectory of physical development

Previous studies have suggested that multiple approaches such as longitudinal mixed-effects and latent growth curve models were used to explore BMI trajectories in childhood, but these methods allow for individual variability and assume that individuals belong to the same underlying population [20, 21]. Group-based trajectory modeling (GBTM) assumes no individual variation in trajectories within groups and describes underlying subgroups or classes within a population based on different trajectories [22].

We used GBTM to fit children’s BMI trajectories (from birth to 24 months and from birth to 72 months of age), head circumference trajectories (from birth to 24 months of age), waist circumference trajectories (from 30 months to 72 months of age), and body fat trajectories (from 48 months to 72 months of age). Children with at least three age-specific physical measurements were allowed to examine quadratic trajectory models. The BMI and head circumference z-scores were used, and raw data on waist circumference and body fat were used to fit the respective trajectories. Latent trajectory patterns of physical growth were identified using GBTM applied to longitudinal data.

**Fig. 2:**
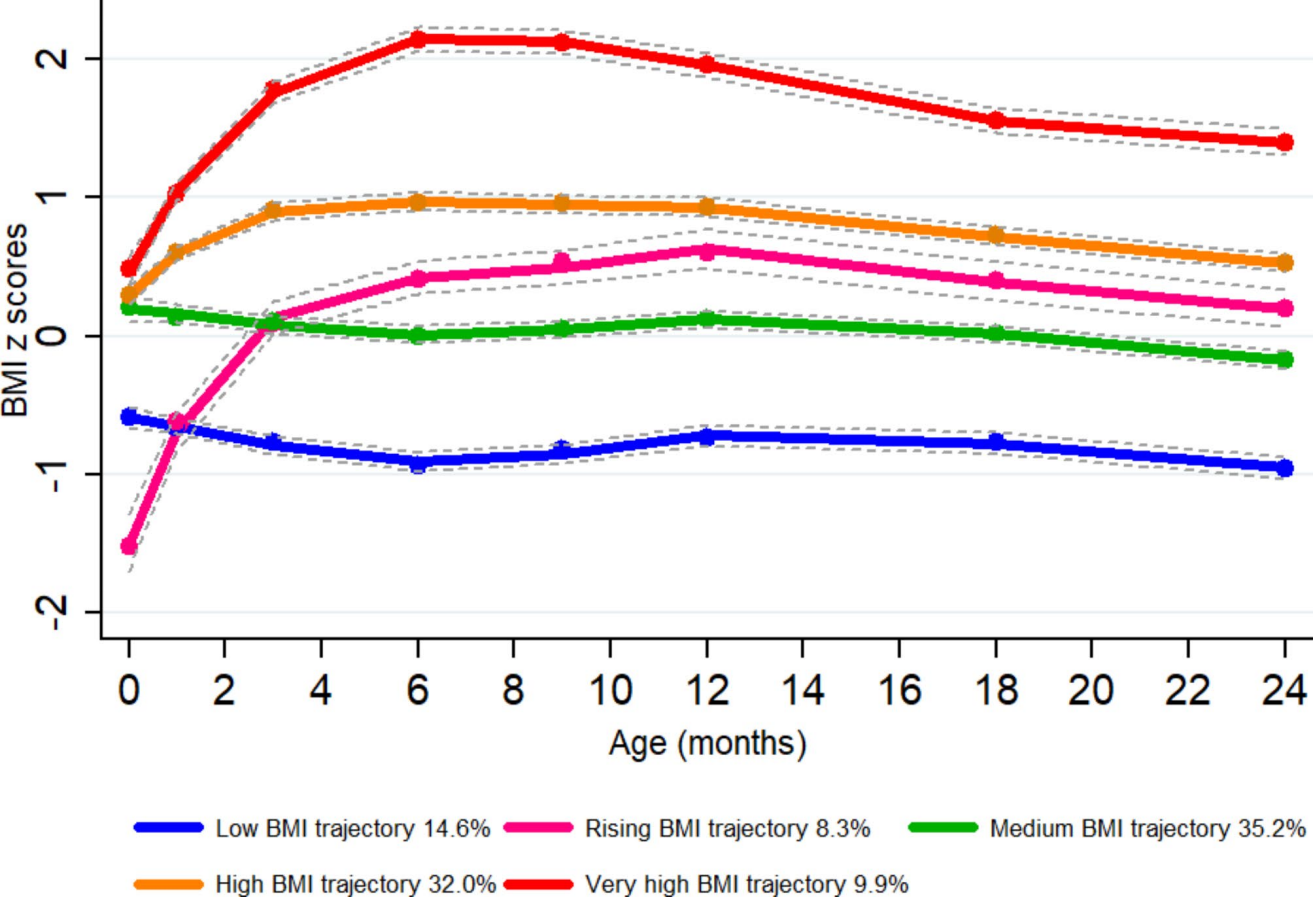
Body mass index (BMI) trajectories of children from birth to 24 months of age

**Fig. 3:**
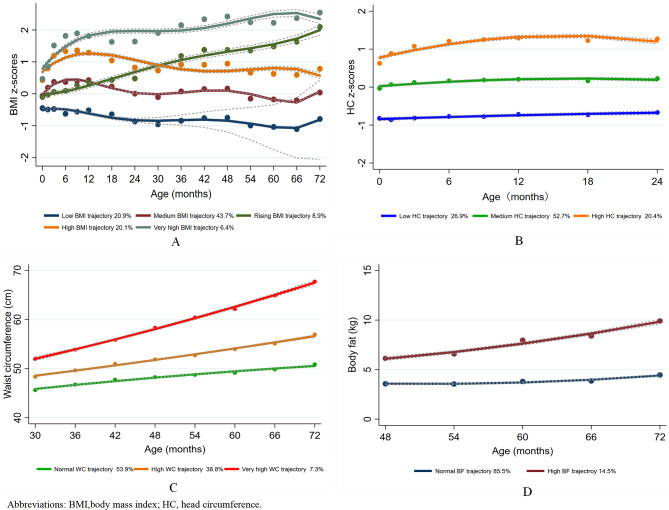
Early childhood physical growth trajectories (A, BMI trajectories of children from birth to 72 months of age; B, Head circumference trajectories of children from birth to 24 months of age; C, waist circumference trajectories of children from 30 to 72 months of age; D, body fat trajectories of children from 48 to 72 months of age)

The maximum likelihood method was adopted for parameter estimation and model fitting. First, by combining existing studies on children’s physical growth trajectories [20], we explored two to six latent classes of trajectory models. Next, we tested statistical significance from the perspective of cubic, quadratic, and linear degrees (that is, from high to low, respectively). If the high degree was not significant, a lower degree was tested. Based on the recommendations of previous studies [22, 23], the detailed selection criteria for the best-fit trajectory model were as follows: (1) Bayesian information criteria (BIC), i.e., the closer the BIC values are to 0, the better the fit of the model; (2) the value of entropy indicated how well the classification distinguished from one group to another; (3) an average posterior probability greater than 0.7; and (4) each trajectory contained at least 5% of participants. Table S1 shows the results of the estimation of the trajectory parameters using GBTM. Finally, five different BMI growth trajectories were determined from birth to 24 months: a low BMI trajectory (14.6%), a rising BMI trajectory (8.3%), a medium BMI trajectory (35.2%), a high BMI trajectory (32.0%), and a very high BMI trajectory (9.9%) (Fig. 2). From birth to 72 months of age, five BMI trajectories were also determined: low (20.9%), rising (8.9%), medium (43.7%), high (20.1%), and very high (6.4%) (Fig. 3A). For head circumference, we identified three trajectories: a low trajectory (26.9%), a medium trajectory (52.7%), and a high trajectory (20.4%) (Fig. 3B). As for waist circumference, we identified three trajectories: a normal trajectory (53.9%), a high trajectory (38.8%), and a very high trajectory (7.3%)] (Fig. 3C). We also identified two body fat trajectories: a normal trajectory (85.5%) and a high trajectory (14.5%) (Fig. 3D).

#### Estimation of AR

Age at AR was defined as the last minimum (nadir) BMI before a sustained increase in BMI, usually occurring at 4–6 years of age [1]. The average number of BMI follow-ups for the included children (n = 2705) from birth to 6 years of age was 13.7 (interquartile range 13–15). Based on the requirement for more data points near the turning points and during periods of rapid change, we set these age intervals and the corresponding minimum numbers of visits [11, 24]. The number of follow-ups in this study was large (16 times in total); in particular, the number of follow-ups in the first year of life was up to five times, which ensured the accuracy of the model fitting. Furthermore, another study by our team obtained a similar median age in AR (4.38 years old) and the prevalence of early AR (39.3%) based on different inclusion criteria (number of BMI measurements > 8) [25]. Given the high number of repeated BMI measurements in this study, to obtain an accurate age in AR, we used the method recommended by Wen et al. [11] to fit the overall BMI trajectory. The fractional polynomial method (using diverse combinations of age terms—Age^p^_j_; powers p_j_ were selected from a fixed set of eight candidate values, including–2, − 1, − 0.5, or log 0.5, 1, 2, and 3) to simulate children’s BMI trajectory as a function of age. The detailed methodology is described in our previous study [26].

We fit the BMI trajectory using a mixed effects model that specified the fixed effects for each fractional polynomial term, which reflected the trend of the population mean and random effects for each term for each child and modeled each child’s deviation from the population mean. We selected the optimal mean and residual variance-covariance structures before model selection based on the Bayesian information criterion (BIC).

We selected the best combination of age ^(−2)^, age ^(−1)^, age ^(−0.5)^, log ^(age)^, and age ^0.5^ by comparing different combinations of fractional polynomial functions that could effectively reflect the BMI trajectory in children 0–6 years of age. From the model, we derived the age at AR for each child and defined children with AR < 48 months as early AR.

### Covariates

We relied on a review of the literature and a directed acyclic graph (DAG) to identify possible confounders, including maternal age [27], family monthly income per capita (< 2500 RMB; 2500–4000 RMB; >4000 RMB) [28], parental education level (junior high school or below; senior middle school; junior college; bachelor’s degree or above) [29], maternal pre-pregnancy BMI [30], paternal BMI [31], parity (multipara; nulliparous) [32], maternal metabolic dysfunctions (yes; no) [33], alcohol use (yes; no) [34], tobacco use (yes; no) [35], maternal iron supplementation (yes; no) [36], maternal folic acid supplementation (yes; no) [37] during pregnancy, and sex of children (male; female) [38] (Figure S1).

Data on maternal age, parental educational level, family income, parity, paternal BMI, alcohol use, tobacco use, iron supplementation, and folic acid supplementation during pregnancy were collected using a questionnaire survey. Maternal weight was measured during recruitment and considered before pregnancy. Information on maternal metabolic dysfunction and parity was extracted from medical notes. Maternal metabolic dysfunction included gestational diabetes and hypertensive disorders during pregnancy, and women with at least one dysfunction were defined as having maternal metabolic dysfunction. Information on alcohol and tobacco use during pregnancy was obtained from maternal and child health records. Furthermore, the data on exclusive breastfeeding for the first 6 months (yes, no), primary caregivers before 3 years (parents; grandparents), screen time (≤ 1 h/day; >1 h/day), and outdoor activity time (≤ 1 h/day; >1 h/day) were reported by the children’s caregivers in the follow-up questionnaire survey. These variables were collected as precision variables for sensitivity analysis.

### Statistical analysis

SPSS (IBM, version 23.0) was used for the statistical analyses. A *P*-value < 0.05 was considered statistically significant. STATA 15.0 was used to fit physical growth trajectories and to estimate children’s AR.

The normality of age-specific distributions of the physical growth data was examined using Q-Q plots and the Kolmogorov-Smirnov test. This showed that BMI, head circumference, waist circumference, and body fat were normally distributed at all age points.

The χ^2^ tests and *t* tests were adopted to evaluate the basic demographic differences between the included and excluded population and the differences in physical values for each exam point between the early AR and non-early AR groups. Poisson regression models (parameter estimation using robust standard errors) were used to examine the association of birth outcomes and early growth patterns (RWG, BMI trajectory, head circumference trajectory, waist circumference trajectory, and body fat trajectory) with early AR. All the above analyses were performed in the crude model (model 1) and the model adjusted for confounders (model 2).

Multiple imputations were used to interpolate missing data from confounders to increase statistical power and validate the study conclusions, and twenty sets of interpolations were created. According to Rubin’s Law [39], the effects were aggregated to obtain new results.

To examine the stability of the results, we performed three sensitivity analyses. Breastfeeding affects physical development in children [40], and other factors in early childhood such as outdoor activity time [41], the main caregiver before 3 years old [42] and screen time [43]) were also strongly associated with early childhood physical development. These variables may be mediating/moderating variables and it is important to do as the sensitivity analysis rather than the main analysis. Therefore, we first further adjusted for exclusive breastfeeding in the first six months based on model 2. Second, we further adjusted for these important covariates (outdoor activity time, main caregiver and screen time) based on model 2. Furthermore, considering that we used the BMI z-score for trajectory fitting, we further used the raw BMI values for trajectory fitting to compare the consistency of the association with the early AR (Figure S4).

## Results

### Basic characteristics of participants

The characteristics of the participants are listed in Table 1. The average maternal age was 26.5 (3.6) years, and the average maternal BMI before pregnancy was 20.9 (2.9) kg/m^2^. Most women were primiparas (90.8%), nonsmokers (99.8%), nonalcohol consumers (92.0%), and had no pregnancy-related complications (82.8%). Among the children, 264 (9.8%) were SGA, 60 (2.2%) had LBW, and 111 (4.1%) were PTB (Table S2). The proportion of children with RWG from birth to 12 months of age, 18 months of age, and 24 months of age was 45.2%, 37.8%, and 37.9%, respectively. The parents of the included children had a higher educational level, and mothers were more likely to be primiparous and have slightly higher gestational weeks.

**Table 1:** Characteristics of included and excluded participants in the study

Characteristics	Included subjects (n = 2705)	Excluded subjects (n = 568)	*P*-value
*Demographic Characteristics*			
Age, years [Mean (SD)]	26.5(3.6)	26.0(3.7)	0.131
Race [n (%)]			0.358
Han	2664(98.5)	556(97.9)	
Others	41(1.5)	12(2.1)	
Parity [n (%)]			0.016
Multipara	250(9.2)	72(12.7)	
Nulliparous	2455(90.8)	496(87.3)	
Maternal educational levels [n (%)]			< 0.001
Junior high school or below	513(19.0)	149(26.2)	
Senior middle school	608(22.5)	125(22.0)	
Junior college	843(31.2)	173(30.5)	
Bachelor degree or above	741(27.4)	121(21.3)	
Incomes (RMB) [n (%)]			0.211
< 2500	718(26.5)	142(25.0)	
2500**–**4000	1144(42.3)	263(46.3)	
> 4000	843(31.2)	163(28.7)	
Maternal pre-pregnancy BMI, kg/m^2^ [Mean (SD)]	20.9(2.9)	20.8(2.6)	0.383
Alcohol use during pregnancy [n (%)]			1.000
No	2489(92.0)	523(92.1)	
Yes	216(8.0)	45(7.9)	
Tobacco use during pregnancy [n (%)]			0.595
No	2700(99.8)	568(100)	
Yes	5(0.2)	0(0)	
Pregnancy complications^a^ [n (%)]			0.466
No	2239(82.8)	463(81.5)	
Yes	466(17.2)	105(18.5)	
Iron supplementation during pregnancy			0.020
No	197(7.3)	58(10.2)	
Yes	2508(92.7)	510(89.8)	
Folic acid supplementation during pregnancy			0.025
No	1531(56.6)	351(61.8)	
Yes	1174(43.4)	217(38.2)	
Father’s educational levels [n (%)]			< 0.001
Junior high school or below	368(13.6)	117(20.6)	
Senior middle school	747(27.6)	159(28.0)	
Junior college	750(27.7)	140(24.6)	
Bachelor degree or above	840(31.1)	152(26.8)	
Father’s BMI	23.4(3.6)	23.3(3.5)	0.542
*Child characteristics*			
Sex [n (%)]			0.926
Female	1326(49.0)	277(48.8)	
Male	1379(51.0)	291(51.2)	
Birth size [Mean (SD)]			
Length, cm	50.04(1.8)	50.0(1.8)	0.406
Weight, g	3366.4(440.4)	3356.5(477.1)	0.631
BMI, kg/m^2^	13.4(1.4)	13.4(1.4)	0.799
Head circumference, cm	34.0(1.6)	30.4(1.4)	0.131
Gestational age, weeks [Mean (SD)]	39.1(1.4)	38.9(1.5)	0.018
Exclusive breastfeeding duration ≥ 6 mo [n (%)]			0.008
Yes	267(9.9)	78(13.7)	
No	2438(90.1)	490(86.3)	
Main caregivers before 3 years [n (%)]			< 0.001
Parents	1376(50.9)	356(62.7)	
Grandparents	1329(49.1)	212(37.3)	
Average screen time per day [n (%)]			0.312
≤ 1 h/day	976(36.1)	192(33.8)	
> 1 h/day	1729(63.9)	376(66.2)	
Average outdoor activity time per day [n (%)]			0.361
≤ 1 h/day	670(24.8)	130(22.9)	
> 1 h/day	2035(75.2)	438(77.1)	

^a^Including gestational diabetes and pregnancy hypertensive disorders

Figure S2 shows an overall plot of the BMI trajectory. Among the included children, 39.9% had AR before the age of 48 months and 16.0% after 72 months (Table S2). The estimated median age in AR was 52 months (4 years and 4 months).

Children with early AR were found to have lower levels of parental education compared to children without early AR. Additionally, mothers of children with early AR had higher rates of pregnancy complications, and these mothers had lower rates of taking folic acid supplements during pregnancy. Furthermore, children with early AR had smaller physical measurements at birth, including weight, length, BMI, and head circumference (Table S3).

### Birth outcomes and AR

Table 2 shows the relationship between birth outcomes and early AR. Children with PTB (vs. non-PTB) (RR = 1.50; 95% CI 1.17 to 1.93), SGA (vs. AGA) (RR = 1.37; 95% CI 1.14 to 1.64), and LBW (vs. ABW) (RR = 1.86; 95% CI 1.28 to 2.51) had increased risk of early AR. Furthermore, children with LGA children (vs. AGA) (RR = 0.79; 95% CI 0.66 to 0.94) and HBW (vs. ABW) (RR = 0.65; 95% CI 0.50 to 0.85) had a lower risk of early AR.

**Table 2:** Associations between birth outcomes and early AR

Birth outcomes		Sample size (n)	Early AR [RR (95%CI)]	
			Model 1	Model 2
Categorical variables				
Birth weight				
	LBW	60	1.92(1.43,2.58)^**^	1.86(1.28,2.51)^**^
	ABW	2430	1.00	1.00
	HBW	215	0.71(0.55,0.92)^**^	0.65(0.50,0.85)^**^
Gestational age				
	PTB	111	1.55(1.21,1.98)^**^	1.50(1.17,1.93)^**^
	NPTB	2594	1.00	1.00
Birth weight by gestational age				
	SGA	264	1.32(1.10,1.58)^**^	1.37(1.14,1.64)^**^
	AGA	2002	1.00	1.00
	LGA	439	0.85(0.71,1.01)	0.79(0.66,0.94)^**^

Abbreviations: PTB, premature birth; NPTB, non-premature birth; HBW, high birth weight; ABW, adequate birth weight; LBW, low birth weight; LGA, large for gestational age; AGA, appropriate for gestational age; SGA, small for gestational age; AR, adiposity rebound

Model 1: Crude model

Model 2: Adjusted for maternal age, family monthly income per capita, maternal education level, paternal education level, maternal pre-pregnancy BMI, paternal BMI, parity, maternal metabolic dysfunctions during pregnancy, alcohol use during pregnancy, tobacco use during pregnancy, iron supplementation during pregnancy, folic acid supplementation during pregnancy, and children’s sex

*: P < 0.05; **: P < 0.01

### Early growth patterns and AR

#### RWG and AR

As shown in Table 3, RWG (from birth to 12 months of age [RR = 1.21; 95% CI 1.07 to 1.37]); from birth to 18 months of age (RR = 1.48; 95% CI 1.30 to 1.67); from birth to 24 months of age (RR = 1.59; 95% CI 1.40 to 1.81]) was significantly associated with an increased risk of early AR in children.

**Table 3:** Associations of RWG and BMI trajectories in the first two years of life and early AR

Physical growth indicators		Sample size (n)	Early AR[RR (95%CI)]	
			Model 1	Model 2
RWG (vs. NRWG)				
*Birth-12 months*				
	RWG	1162	1.22(1.08,1.38)^**^	1.21(1.07,1.37)^**^
	Non-RWG	1406	1.00	1.00
*Birth-18 months*				
	RWG	971	1.48(1.31,1.67)^**^	1.48(1.30,1.67)^**^
	Non-RWG	1598	1.00	1.00
*Birth-24 months*				
	RWG	930	1.60(1.41,1.82)^**^	1.59(1.40,1.81)^**^
	Non-RWG	1524	1.00	1.00
**BMI trajectories**				
*Birth-24 months*				
	Low BMI trajectory	395	1.22(1.01,1.46)^*^	1.27(1.06,1.53)^**^
	Rising BMI trajectory	225	1.52(1.24,1.87)^**^	1.50(1.22,1.84)^**^
	Medium BMI trajectory	951	1.00	1.00
	High BMI trajectory	866	0.98(0.84,1.14)	0.96(0.82,1.11)
	Very high BMI trajectory	268	1.17(0.95,1.45)	1.04(0.84,1.28)
**Head circumference trajectories**				
*Birth-24 months*				
	Low head circumference trajectory	728	1.09(0.93,1.28)	1.12(0.97,1.28)
	Medium head circumference trajectory	1425	1.00	1.00
	High head circumference trajectory	552	1.18(0.99,1.41)	0.93(0.79,1.09)

Abbreviations: BMI, body mass index; RWG, rapid weight gain; NRWG, no rapid weight gain; AR, adiposity rebound

Model 1: Crude model

Model 2: Adjusted for maternal age, family monthly income per capita, maternal education level, paternal education level, maternal pre-pregnancy BMI, paternal BMI, parity, maternal metabolic dysfunctions during pregnancy, alcohol use during pregnancy, tobacco use during pregnancy, iron supplementation during pregnancy, folic acid supplementation during pregnancy, and children’s sex

*: P < 0.05; **: P < 0.01

#### BMI trajectory and AR

Compared to non-EAR, the BMI of children with early AR tended to be initially lower (from birth to 6 months) and then higher (from 18 to 72 months). In particular, from the age of 18 months (mean difference = 0.3 kg/m^2^) to the age of 72 months (mean difference = 1.6 kg/m^2^), the difference in mean BMI between children with early AR and non-early AR showed a gradual increase (Figure S3). The detailed data are shown in Table S4.

For the BMI trajectory from birth to 24 months of age, we found that a significantly higher proportion of early AR occurred between the low BMI trajectory (44.7%) and the increasing BMI trajectory (56.0%) compared to the medium BMI trajectory (36.8%). For the BMI trajectory of children from birth to 72 months of age, we found that a significantly higher proportion of early AR occurred on the increasing BMI trajectory (99.6%) and the high BMI trajectory (90.8%) than in the medium BMI trajectory (30.2%) (Table S5).

After adjusted the confounders, it was observed that children with a low BMI trajectory (RR = 1.27; 95% CI 1.06 to 1.53) and a rising BMI trajectory (RR = 1.50; 95% CI 1.22 to 1.84) in the first two years of life had a higher risk of early AR than those with a medium-level trajectory (Table 3). Children with a very high BMI trajectory (RR = 2.83; 95% CI 2.33 to 3.43) and a rising BMI trajectory (RR = 3.15; 95% CI 2.66 to 3.72) from birth to 72 months of age both increased the risk of early AR (Table 4).

**Table 4:** Associations of the trajectories of physical growth and early AR in children

Physical growth indicators		Sample size (n)	Early AR[RR (95%CI)]	
			Model 1	Model 2
**Waist circumference**				
*30–72 months*				
	Normal waist circumference trajectory	1456	1.00	1.00
	High waist circumference trajectory	1035	2.58(2.24,2.96)^**^	2.52(2.19,2.90)^**^
	Very high waist circumference trajectory	193	4.47(3.73,5.37)^**^	4.17(3.43,5.06)^**^
**Body fat**				
*48–72 months*				
	Normal body fat trajectory	1610	1.00	1.00
	High body fat trajectory	578	3.20(2.80,3.66)^**^	3.01(2.62,3.46)^**^
**BMI z scores**				
*Birth-72 months*				
	Low BMI trajectory	570	0.76(0.62,0.92)^**^	0.78(0.63,0.95)^*^
	Rising BMI trajectory	241	3.30(2.80,3.89)^**^	3.15(2.66,3.72)^**^
	Medium BMI trajectory	1183	1.00	1.00
	High BMI trajectory	541	1.21(1.01,1.44)^*^	1.18(0.99,1.40)
	Very high BMI trajectory	174	3.00(2.50,3.63)^**^	2.83(2.33,3.43)^**^

Abbreviations: early AR, early adiposity rebound; BMI, body mass index

Model 1: Crude model

Model 2: Adjusted for maternal age, family monthly income per capita, maternal education level, paternal education level, maternal pre-pregnancy BMI, paternal BMI, parity, maternal metabolic dysfunctions during pregnancy, alcohol use during pregnancy, tobacco use during pregnancy, iron supplementation during pregnancy, folic acid supplementation during pregnancy, and children’s sex

*: P < 0.05; **: P < 0.01

#### Head circumference trajectory and AR

Compared to children with non-EAR, children with early AR had a lower trajectory of head circumference from birth to 9 months of age, a similar trajectory of head circumference from 12 to 18 months of age, and finally a higher trajectory of head circumference at 24 months of age (Figure S3). The detailed data are shown in Table S4.

Despite subtle differences in head circumference between children with early AR and children with non-early AR, we did not find a significant association between head circumference trajectories and early AR in the logistic regression (Table 3).

#### Body fat trajectory and AR

In children aged 48 to 72 months, mean body fat values were significantly higher in the early AR group than in the non-early AR group (Figure. S3). The detailed data are shown in **Table S4**. A significantly higher proportion of early AR was observed in children with a high body fat trajectory (79.9%) than in those with a normal body fat trajectory (25.0%) (Table S5).

After adjusted the confounders, we found that children with a high body fat trajectory was associated with an elevated risk of early AR (RR = 3.01; 95% CI 2.62 to 3.46) (Table 4).

#### Waist circumference trajectory and AR

In children aged 30 to 72 months, mean waist circumference values were significantly higher in the early AR group than in the non- early AR group (Figure. S3). The detailed data are shown in Table S4 A significantly higher proportion of early AR was observed in the high- waist circumference trajectory (55.2%) and very high waist circumference trajectory (95.9%) than in the normal waist circumference trajectory (21.4%) (Table S5).

After adjusted the confounders, we found that children with a high body fat trajectory (RR = 2.52; 95% CI 2.19 to 2.90) and very high body fat trajectory (RR = 4.17; 95% CI 3.43 to 5.06) were associated with an elevated risk of early AR (Table 4).

### Sensitivity analyses

The sensitivity analyses did not fundamentally change the main results (Tables S6 to S9).

## Discussion

We comprehensively assessed the association of early childhood physical growth trajectories, particularly growth patterns in the first two years of life, with early AR. Our findings support a positive association of overall very high BMI, high waist circumference and high body fat trajectories in early childhood with a higher risk of early AR in childhood. Moreover, the pattern of physical growth in the first two years of a child’s life showed a particular association with early AR. In detailed, children born to PTB, SGA and LBW had a higher risk of early AR; children with RWG, rising BMI trajectory and low BMI trajectory from birth to 24 months all related to an increased risk of early AR.

We found that a high BMI trajectory from birth to 72 months was significantly associated with early AR in children. These findings were consistent with the current main findings that children at a younger age at AR have a higher BMI and are at higher risk of being overweight and obesity [5, 44]. We also found that a high waist circumference trajectory and body fat trajectory were significantly associated with early AR, especially in children with very high waist circumference and body fat trajectories, with early AR rates of up to 95.6% and 96.8%, respectively. This suggests that the strong association between the early onset of AR and high BMI may be related to the fat mass index (FMI). It is worth noting that the BMI trajectory of children birth to 24 months of age showed a unique association with early AR. Children with a low BMI trajectory and an increasing BMI trajectory in the first two years of life had a higher risk of experiencing early AR than those with a medium BMI trajectory. Children with a low BMI trajectory are more likely to experience RWG after birth, but they also had difficulties fully catching up to the level of normal children, and thus there may remain a low trajectory in their first year [45]. Two previous studies also indirectly supported our findings that children with early AR had a low BMI before AR [6, 7]. Furthermore, we compared the BMI levels of children with early AR and non-early AR at each age and found that children with early AR had a lower BMI from birth to 6 months, but a higher BMI from 18 to 72 months. The two categories of BMI trajectories (low trajectory and rising trajectory) were associated with early AR; however, the possible metabolic risk later needs to be considered in the context of changes in body composition. BMI includes both the components of FMI and lean mass index (LML), while the appearance of AR (the second rise in adiposity) corresponds to the beginning of an increase in the number of fat cells after an early phase of increasing and then decreasing in size [46], which represents an overall cessation of the decline in FMI [47]. Rolland-Cachera et al. [48] proposed two main growth trajectories associated with adult obesity: a high BMI for all ages, reflecting both high lean body mass and high-fat body mass, and a low or normal BMI, followed by an early AR reflecting increased fat mass rather than lean body mass. Early rapid growth patterns can be detrimental if they correspond to catch-up growth, but they can also correspond to a more favorable outcome if they correspond to increased LML. Rapid increases in BMI before the age of 2 years have been shown to increase LML in adults without excess fat accumulation, while rapid increases in BMI in late childhood result in relatively large increases in fat mass despite a concomitant increase in LML [49].

The mean age at AR was earlier for the children in current study. The proportions of early AR (age at AR < 48 months) and AR < 5 years were 39.9% and 66.9%, respectively. This phenomenon is not surprising, as there is a general trend toward an earlier age for AR in children [5]. The Korean National Health Information Database (2008–2015), also located in East Asia, found that the median time point for AR was 45 months and the proportion of patients with AR before 4.75 years was as high as 79.6% [50]. Because of the differences in physical development of children in different countries and regions, there are currently no uniform standards for early AR. Consequently, most of the criteria for Rolland-Cachera et al. [1] were adopted, as he was the first to propose the concept of AR. Our group’s systematic review of all published AR-related studies found that the overall prevalence of early AR according to Rolland-Cachera et al.’s definition is 40% [5]. Combined with the age cutoff value of the first 40% of children in this study, this is almost consistent with 48 months of age as a criterion for defining early AR. This criterion has also been used in other studies [10, 25].

Our results indicated that children born to PTB, SGA and LBW all associated with early AR. Researchers in the EDEN cohort found that the risk of early AR was higher in children with SGA than in children with AGA, but no differences in early AR were found between children with LGA and AGA or between children with PBT and non-PTB [9]. Baldassarre et al. [51], based on a small cohort study of preterm infants, found that PTB was an independent risk factor for late AR. This difference may be due to different definitions of early AR and different study populations. The study by Baldassarre et al. [9] was limited to premature infants and early AR was defined as being in the lowest quintile of age in AR in the EDEN cohort, which could have contributed to the observed differences. Numerous population-based observational studies have shown that children with SGA, LBW, and PTB undergo rapid growth in early childhood [52–54]. Our study found that children with RWG in the first two years were more likely to experience early AR. The phenomenon of AR is that the BMI starts to increase, whereas the essence is that the fat mass index (FMI) stops declining [47]. Studies have found that infants with RWG between birth and 2 years of age have higher FMI at 2 years of age than those without RWG [55]. Focusing on trends in both BMI and FMI may provide further insight into early childhood physical development. The RWG and early AR are associated with later obesity and metabolic risk, suggesting the importance of focusing on early physical growth patterns to prevent and control long-term obesity. A study by Koyama et al. [8] found no association between early weight gain and the timing of AR, possibly because this study did not use a definition of sustained early RWG (from birth to 12 months or 24 months) but instead adopted increments every four months of weight gain (from birth to 4 months, 4–8 months, and 8–12 months), which can underestimate the association between RWG and early AR.

In this study, the GBTM was used to fit trajectories to children’s repeated measures of physical indicators. Existing studies have demonstrated that both GBTM and growth mixture modelling are suitable for modeling trajectories [56], and although both methods have different approaches to longitudinal noble modeling, studies have observed strong agreement on the optimal number of trajectories su and have recommended the use of GBTM because it is less computationally intensive [57]. The strengths of GBTM for fitting somatic growth indicators have been reported and adopted in a number of studies, primarily because it allows researchers to identify and characterize potential subgroups or categories within populations based on different trajectories [22].

The age of AR in children is getting younger and upstream factors such as prenatal or early life factors may be important contributors to its variability. Existing studies suggest that limited maternal education, low socioeconomic status, increased maternal BMI, gestational diabetes, and increased caloric intake are significant predictors of early AR [9, 58–60]. Previous studies by our group have also found that maternal pre-pregnancy BMI and gestational diabetes have independent and combined effects on the timing of early AR [25], and elective cesarean section increases the risk of early AR in girls [26]. There are fewer other studies from Chinese birth/children cohorts, and only one longitudinal study from Shanghai reported that older mothers, shorter duration of breastfeeding, and SGA were associated with a higher risk of developing early AR [10]. Some studies have shown that increasing the duration of breastfeeding, rationalizing protein intake, and reducing processed and processed foods are possible diet interventions that delay the early onset of AR [61, 62].

It needs to be recognized that both parents of the included children had a higher educational level, with mothers having higher rates of iron supplementation and folic acid supplementation during pregnancy as well as longer gestational weeks compared to the non-included children. Thus, potential selection bias may exist. Compared with children who did not have early AR, children with early AR had both parents with lower educational levels, a higher proportion of mothers with pregnancy complications during pregnancy, a lower proportion of mothers with folic acid supplementation during pregnancy, and smaller physical measures at birth including weight, length, BMI, and head circumference. Although we controlled for multiple important confounders during pregnancy and at birth, the above differences also suggest that factors affecting maternal pregnancy complications and birth outcomes may be important residual confounders.

This study has several strengths. This is a prospective birth cohort study to explore the relationship between early growth trajectories and the timing of AR, and all information on exposure, outcome and potential confounders was collected prospectively. Second, the high frequency of anthropometric measurements, with up to 16 repetitions of BMI measurements from birth to 6 years, provided a realistic prediction of age at AR. Third, we considered other postnatal factors affecting a child’s physical development, such as breastfeeding, primary caregivers, and screen time and outdoor activities time. These factors are not confounders between exposure and outcome but are important precision variables. The inclusion of these variables is expected to improve the precision of our findings.

Several limitations need to be acknowledged. First, we only have follow-up data for children up to age six years. According to the model, 13.2% of children did not undergo AR. Longer follow-up periods are required to determine the age of AR in these children. Second, although we collected information prospectively, we could not avoid some recall/reporting biases, such as maternal smoking, outdoor activity time, and exclusive breastfeeding. However, the use of repeated data collection in this cohort largely reduced the unreliability of reporting. Third, there are some drivers in early childhood that may influence physical development have not been collected, such as in-home nurturance, child feeding, diet, and sleep quality, stimulation, and some family and school environment factors. Therefore, we cannot further explore the potential modifying effects of these important early childhood covariates. Fourth, smaller numbers of low and high birth weights may increase the probability that the association is a false positive. However, the associations of SGA and LGA with outcome showed consistent direction and close association strength, which suggests the reliability of our findings regarding HBW and LBW with outcome.

## Conclusions

This study showed that children with high BMI, waist circumference, and body fat trajectories in early childhood had a high prevalence of early AR. Infants born to SGA, LBW and PTB were at higher risk for early AR. Furthermore, children with a low head circumference trajectory, low BMI trajectory, rising BMI trajectory, and RWG in the first two years of life were associated with an increased risk of early AR. The effect of early life physical developmental indicators, especially BMI, on early AR appears to be in different directions of association before and after 12 months of age. These results can help to understand the early factors and processes that lead to metabolic risks.

### Electronic supplementary material

Below is the link to the electronic supplementary material.

**Supplementary Material 1**: Table S1 Results of group-based trajectory model. Table S2 Age at AR in children with different birth outcomes. Table S3 The basic characteristics of participants with and without early AR. Table S4 Physical growth levels of children in the early AR and non-early AR groups at each age. Table S5 Proportions of EAR and NEAR in children with different physical trajectories. Table S6 Sensitivity analysis on the association between birth outcomes and early AR. Table S7 Sensitivity analysis on the association of RWG, BMI trajectories and head circumference trajectories with early AR. Table S8 Sensitivity analysis on the association between the trajectories of physical growth and early AR in children. Table S9 Associations of BMI trajectories (using raw BMI data) and early AR in children. Figure S1 Directed acyclic graph of the relationship between birth outcome/early growth patterns and age at AR. Figure S2 The overall trajectory plot based on the BMI fit of 2705 children. Figure S3 Physical growth trajectory of children in the early AR (EAR) and non-early AR (NEAR) groups. Figure S4 Trajectories fitted using raw BMI data.

## Data Availability

Because this data was derived from the Ma’anshan birth cohort, the datasets generated and/or analyzed in the current study are not publicly available, but are available from the corresponding authors upon reasonable request.

## Abbreviations

AR: adiposity rebound
BMI: body mass index
RWG: rapid weight gain
GBTM: group-based trajectory modeling
SGA: small for gestational age
PTB: preterm birth
LBW: low birth weight
WAZ: Weight-for-age z-score
BMIZ: BMI-for-age z-score
WHO: World Health Organization
BIC: Bayesian information criterion
DAG: directed acyclic graph
MABC: Ma’anshan Birth Cohort Study
