# Supplementary material for: Birth outcomes and early growth patterns associated with age at adiposity rebound: the Ma’anshan birth cohort (MABC) study
Source: BMC Public Health. 2023 Dec 4;23:2405. doi: 10.1186/s12889-023-17236-9 (PMC10694931; doi:10.1186/s12889-023-17236-9)
Supplement: Supplementary file 1 — Supplementary Material 1: Table S1 Results of group-based trajectory model. Table S2 Age at AR in children with different birth outcomes. Table S3 The basic characteristics of participants with and without early AR. Table S4 Physical growth levels of children in the early AR and non-early AR groups at each age. Table S5 Proportions of EAR and NEAR in children with different physical trajectories. Table S6 Sensitivity analysis on the association between birth outcomes and early AR. Table S7 Sensitivity analysis on the association of RWG, BMI trajectories and head circumference trajectories with early AR. Table S8 Sensitivity analysis on the association between the trajectories of physical growth and early AR in children. Table S9 Associations of BMI trajectories (using raw BMI data) and early AR in children. Figure S1 Directed acyclic graph of the relationship between birth outcome/early growth patterns and age at AR. Figure S2 The overall trajectory plot based on the BMI fit of 2705 children. Figure S3 Physical growth trajectory of children in the early AR (EAR) and non-early AR (NEAR) groups. Figure S4 Trajectories fitted using raw BMI data. [file 12889_2023_17236_MOESM1_ESM.pdf]

Table S1 Results of group-based trajectory model.

Physical trajectory		Latent classes	Polynomial degree	BIC	AIC	Log-Likelihood	% Participants per class	Entropy
BMI trajectory								
	0-24 months							
		2	Cubic	-22452.92	-22428.74	-22420.74	54.4/45.6	0.745
		3	Cubic	-21711.78	-21675.50	-21663.50	25.9/56.5/17.6	0.778
		4	Cubic	-21394.26	-21345.90	-21329.90	23.5/10.1/49.1/17.4	0.780
		**5**	**Cubic**	**-21139.87**	**-21079.42**	**-21059.42**	**14.6/8.3/35.2/32.0/9.9**	**0.773**
		6	Cubic	-20986.73	-20914.19	-20890.19	12.9/4.5/18.4/37.6/18.8/7.9	0.746
	0-72 months							
		2	Cubic	-53278.71	-53254.40	-53246.40	56.6/43.4	0.869
		3	Cubic	-50870.76	-50834.30	-50822.30	30.9/52.5/16.6	0.880
		4	Cubic	-49949.12	-49900.51	-49884.51	17.3/41.1/31.5/10.1	0.842
		**5**	**Cubic**	**-49288.88**	**-49228.11**	**-49208.11**	**20.9/43.7/8.9/20.1/6.4**	**0.844**
		6	Cubic	-48791.57	-48718.65	-48694.65	8.4/12.3/27.8/32.1/15.1/4.3	0.829
BF trajectory	48-72 months							
		**2**	**Cubic**	**-16986.58**	**-16963.64**	**-16955.64**	**85.5/14.5**	**0.940**
		3	Cubic	-16301.18	-16266.77	-16254.77	72.8/22.8/4.4	0.904
		4	Cubic	-16032.77	-15986.88	-15970.88	9.6/27.4/60.2/2.8	0.842
		5	Cubic	-15828.26	-15770.90	-15750.90	10.6/3.2/57.1/28.9/0.2	0.854
		6	Cubic	-15756.76	-15687.93	-15663.93	14.9/2.4/6.1/41.1/35.3/0.2	0.786
WC trajectory	30-72 months							
		2	Cubic	-43967.37	-43943.48	-43935.48	81.4/18.7	0.920
		**3**	**Cubic**	**-42421.50**	**-42385.66**	**-42373.66**	**53.8/38.8/7.3**	**0.859**
		4	Cubic	-41767.25	-41719.48	-41703.48	37.0/43.8/14.8/4.4	0.832
		5	Cubic	-41500.15	-41440.43	-41420.43	33.3/16.3/43.5/5.5/1.4	0.840

		6	Cubic	-41333.27	-41261.60	-41237.60	11.2/32.3/37.5/12.7/5.0/1.3	0.794
HC trajectory	0-24 months							
		2	Cubic	-26007.41	-25983.24	-25975.24	55.3/44.7	0.842
		**3**	**Cubic**	**-24471.42**	**-24435.17**	**-24423.17**	**26.9/52.7/20.4**	**0.849**
		4	Cubic	-23812.06	-23763.72	-23747.72	12.9/41.3/35.4/10.4	0.838
		5	Cubic	-23402.73	-23342.30	-23322.30	12.2/29.3/14.2/34.0/10.3	0.814
		6	Cubic	-23040.82	-22968.31	-22944.31	9.3/7.4/29.7/24.9/20.4/8.3	0.812

BIC, Bayesian Information Criterion; *AIC*, Akaike's information criteria; The best fitting model is highlighted in bold characters.

Table S2 Age at AR in children with different birth outcomes.

Birth outcomes		Sample size (n)	Age at AR [n (%)]		
			< 48 months	48 ~ 72 months	≥ 72 months
**Total**		2705	1079(39.9)	1194(44.1)	432(16.0)
Birth weight					
	LBW	60	46(76.7)	12(20.0)	2(3.3)
	ABW	2430	972(40.0)	1084(44.6)	374(15.4)
	HBW	215	61(28.4)	98(45.6)	56(26.0)
Gestational age					
	PTB	111	67(60.4)	34(30.6)	10(9.0)
	NPTB	2594	1012(39.0)	1160(44.7)	422(16.3)
Birth weight by gestational age					
	SGA	264	138(52.3)	103(39.0)	23(8.7)
	AGA	2002	793(39.6)	903(45.1.7)	306(15.3)
	LGA	439	148(33.7)	188(42.8.3)	103(23.5)

Abbreviations: PTB, premature birth; NPTB, non-premature birth; HBW, high birth weight; ABW, adequate birth weight; LBW, low birth weight; LGA, large for gestational

age; AGA, appropriate for gestational age; SGA, small for gestational age; AR, adiposity rebound.

Table S3 The basic characteristics of participants with and without early AR.

Characteristics	Children with early AR		P-value
	**Yes (n=2327)**	**No (n=804)**	
Demographic Characteristics			
Age, years [Mean (SD)]	26.4(3.7)	26.5(3.6)	0.565
Race [n (%)]			0.749
Han	1064(98.6)	1600(98.4)	
Others	15(1.4)	26(1.6)	
Parity [n (%)]			0.684
Multipara	103(9.5)	147(9.0)	
Nulliparous	976(90.5)	1479(91.0)	
Maternal educational levels [n (%)]			0.009
Junior high school or below	222(20.6)	291(17.9)	
Senior middle school	254(23.5)	354(21.8)	

Junior college	345(32.0)	498(30.6)	
Bachelor degree or above	258(23.9)	483(29.7)	
Incomes (RMB) [n (%)]			0.201
<2500	91(8.4)	130(8.0)	
2500-4000	530(49.1)	749(46.1)	
>4000	458(42.4)	747(45.9)	
Maternal pre-pregnancy BMI, kg/m^2 [Mean (SD)]			
Alcohol use during pregnancy [n (%)]			0.665
No	996(92.3)	1493(91.8)	
Yes	83(7.7)	133(8.2)	
Tobacco use during pregnancy [n (%)]			0.654
No	1078(99.9)	1622(99.8)	
Yes	1(0.1)	4(0.2)	

Pregnancy complications# [n (%)]			0.001
No	860(79.7)	1379(84.8)	
Yes	219(20.3)	247(15.2)	
Iron supplementation during pregnancy			0.152
No	89(8.2)	109(6.7)	
Yes	993(91.8)	1518(93.3)	
Folic acid supplementation during pregnancy			0.020
No	643(58.4)	892(54.8)	
Yes	439(40.6)	735(45.2)	
Paternal educational levels [n (%)]			0.002
Junior high school or below	164(15.2)	206(12.7)	
Senior middle school	320(29.7)	427(26.3)	
Junior college	303(28.1)	447(27.5)	
Bachelor degree or above	292(27.1)	546(33.6)	

Paternal BMI	24.0(3.5)	23.0(3.6)	<0.001
Child characteristics			
Sex [n (%)]			0.388
Female	540(50.0)	786(48.3)	
Male	539(50.0)	840(51.7)	
Birth size [Mean (SD)]			
Length, cm	49.9(1.9)	50.1(1.7)	<0.001
Weight, g	3295.9(466.9)	3413(415.6)	<0.001
BMI, kg/m^2	13.2(1.3)	13.6(1.4)	<0.001
Head circumference, cm	33.9(1.5)	34.2(1.6)	<0.001
Gestational age, weeks [Mean (SD)]	39.0(1.5)	39.1(1.2)	0.011
Exclusive breastfeeding duration ≥ 6 mo [n (%)]			0.510
Yes	101(9.4)	166(10.2)	

No	978(90.6)	1460(89.8)	
Main caregivers before 3 years [n (%)]			0.530
Parents	539(50.0)	835(51.4)	
Grandparents	540(50.0)	791(48.6)	
Time spent in screening [n (%)]			0.005
≤ 1h/day	352(32.6)	618(38.0)	
> 1h/day	727(67.4)	1008(62.0)	
Time spent in outdoor activities [n (%)]			0.292
≤ 1h/day	25023.2)	406(25.0)	
> 1h/day	829(76.8)	1220(76.8)	

Table S4 Physical growth levels of children in the early AR and non-early AR groups at each age.

Physical growth indicators (Age)	Early AR		Non-early AR		P-value†
	Sample size (n)	Mean (SD)	Sample size (n)	Mean (SD)	
BMI, kg/m^2					
Birth	1616	13.6(1.4)	1069	13.2(1.3)	<0.001
1.4 months	1555	15.9(1.3)	1025	15.5(1.3)	<0.001
3 months	1555	17.5(1.5)	1035	17.1(1.5)	<0.001
6 months	1556	18.0(1.6)	1044	17.7(1.7)	<0.001
9 months	1504	17.8(1.5)	994	17.7(1.5)	0.082
12 months	1537	17.3(1.4)	1028	17.3(1.4)	0.337
18 months	1549	16.3(1.2)	1019	16.6(1.3)	<0.001
24 months	1480	15.8(1.1)	972	16.4(1.5)	<0.001
30 months	1448	15.5(1.1)	952	16.4(1.5)	<0.001
36 months	1533	15.5(1.1)	1019	16.6(1.5)	<0.001
42 months	1259	15.4(1.2)	862	16.9(1.7)	<0.001
48 months	1315	15.3(1.2)	890	17.0(2.0)	<0.001

54 months	1195	14.8(1.0)	782	16.6(1.8)	<0.001
60 months	1128	14.8(1.1)	714	16.7(1.8)	<0.001
66 months	1154	14.7(1.1)	697	17.0(2.0)	<0.001
72 months	937	15.0(1.2)	613	17.6(2.2)	<0.001
Head circumference, cm					
Birth	1544	34.2(1.6)	1029	33.9(1.5)	<0.001
1.4 months	1554	37.9(1.0)	1024	37.6(1.1)	<0.001
3 months	1553	40.3(1.1)	1033	40.1(1.1)	<0.001
6 months	1558	43.0(1.2)	1041	42.9(1.2)	<0.001
9 months	1504	44.7(1.1)	992	44.6(1.3)	0.006
12 months	1520	45.8(1.2)	1014	45.8(1.3)	0.579
18 months	1516	47.0(1.2)	1005	47.1(1.3)	0.101
24 months	1447	48.0(1.2)	944	48.1(1.3)	0.013
Waist circumference, cm					
30 months	1134	46.6(2.8)	756	48.1(3.6)	<0.001
36 months	1449	47.6(2.7)	962	49.5(3.6)	<0.001
42 months	1258	48.7(2.6)	860	50.9(3.8)	<0.001

48 months	1314	49.2(2.7)	890	52.0(4.2)	<0.001
54 months	1195	49.7(2.7)	782	53.4(2.6)	<0.001
60 months	1128	50.2(3.6)	714	54.6(5.2)	<0.001
66 months	1154	50.9(3.3)	697	56.2(5.5)	<0.001
72 months	935	52.1(3.5)	611	58.4(6.4)	<0.001
Body fat, kg					
48 months	1302	3.5(1.4)	884	4.7(1.8)	<0.001
54 months	1194	3.4(1.2)	778	4.9(2.0)	<0.001
60 months	1125	3.7(1.3)	711	5.6(2.6)	<0.001
66 months	1153	3.6(1.2)	695	6.0(2.6)	<0.001
72 months	917	4.2(1.5)	600	7.0(3.0)	<0.001

Abbreviations: BMI: body mass index; early AR: early adiposity rebound.

†: T-test for early AR group vs. non-early AR group.

Table S5 Proportions of EAR and NEAR in children with different physical trajectories.

Physical growth indicators			Early AR	Non-early AR
			n (%)	n (%)
**BMI**				
	Birth-24 months			
		Low trajectory	176(44.7)	217(55.3)
		Rising trajectory	126(56.0)	99(44.0)
		Medium trajectory	350(36.8)	601(63.2)
		High trajectory	311(35.9)	555(64.1)
		Very high trajectory	116(43.1)	152(56.9)
	Birth-72 months			
		Low trajectory	130(22.8)	440(77.2)
		Rising trajectory	240(99.6)	1(0.4)
		Medium trajectory	356(30.2)	825(69.8)
		High trajectory	197(36.4)	344(63.6)
		Very high trajectory	157(91.3)	15(8.7)
**Head Circumference**				

0-24 months			
	Low trajectory	313(43.2)	413(56.9)
	Medium trajectory	566(38.8)	864(61.2)
	High trajectory	202(36.6)	350(63.4)
**Body fat**			
48-72 months			
	High trajectory	461(73.9)	116(20.1)
	Normal trajectory	401(24.9)	1208(75.1)
**Waist circumference**			
36-72 months			
	Very high trajectory	185(95.9)	8(4.1)
	High trajectory	571(55.2)	464(44.8)
	Normal trajectory	312(21.4)	1144(78.6)

Abbreviations: BMI, body mass index; Early AR, early adiposity rebound.

Table S6 Sensitivity analysis on the association between birth outcomes and early AR.

**Birth outcomes**		**Sensitivity analysis 1**	**Sensitivity analysis 2**
		**Early AR [RR (95%CI)]**	**Early AR [RR (95%CI)]**
**Categorical variables**			
Birth weight			
	LBW	1.86(1.37,2.51)**	1.88(1.39,2.53)**
	ABW	1.00	1.00
	HBW	0.65(0.50,0.85)**	0.65(0.50,0.84)**
Gestational age			
	PTB	1.50(1.16,1.93)**	1.49(1.16,1.92)**
	NPTB	1.00	1.00
Birth weight by gestational age			
	SGA	1.37(1.14,1.64)**	1.37(1.14,1.64)**
	AGA	1.00	1.00
	LGA	0.79(0.66,0.94)**	0.78(0.65,0.94)**

Abbreviations: PTB, premature birth; NPTB, non-premature birth; HBW, high birth weight; ABW, adequate birth weight; LBW, low birth weight; LGA, large for gestational age; AGA, appropriate for gestational age; SGA, small for gestational age; Early AR, early adiposity rebound.

Sensitivity analysis 1: adjusted for maternal age, family monthly income per capita, maternal education level, paternal education level, maternal pre-pregnancy BMI, paternal BMI, parity, maternal metabolic dysfunctions during pregnancy, alcohol use during pregnancy, tobacco use during pregnancy, iron supplementation during pregnancy, folic acid supplementation during pregnancy, children's sex, and exclusive breastfeeding duration ≥6 months.

Sensitivity analysis 2: adjusted for maternal age, family monthly income per capita, maternal education level, paternal education level, maternal pre-pregnancy BMI, paternal BMI, parity, maternal metabolic dysfunctions during pregnancy, alcohol use during pregnancy, tobacco use during pregnancy, iron supplementation during pregnancy, folic acid supplementation during pregnancy, children's sex, main caregivers before 3 years, screen time, and outdoor activity time.

*: $P < 0.05$; **: $P < 0.01$.

Table S7 Sensitivity analysis on the association of RWG, BMI trajectories and head circumference trajectories with early AR.

Physical growth indicators		Sensitivity analysis 1	Sensitivity analysis 2
Range of ages	Classification	Early AR [RR (95%CI)]	Early AR [RR (95%CI)]
**RWG**			
Birth-12 months			
	RWG	1.21(1.07,1.37)**	1.21(1.07,1.37)**
	Non-RWG	1.00	1.00
Birth-18 months			
	RWG	1.47(1.30,1.67)**	1.48(1.31,1.67)**
	Non-RWG	1.00	1.00
Birth-24 months			
	RWG	1.59(1.40,1.81)v	1.59(1.40,1.81)**
	Non-RWG	1.00	1.00
**BMI trajectories**			
Birth-24 months			

	Low trajectory	1.27(1.06,1.53)**	1.28(1.07,1.54)**
	Rising trajectory	1.50(1.23,1.85)**	1.49(1.22,1.83)**
	Medium trajectory	1.00	1.00
	High trajectory	0.96(0.82,1.11)	0.95(0.82,1.11)
	Very high trajectory	1.05(0.85,1.30)	1.04(0.84,1.28)
**Head circumference trajectories**			
Birth-24 months			
	Low trajectory	1.12(0.97,1.28)	1.12(0.97,1.29)
	Medium trajectory	1.00	1.00
	High trajectory	0.93(0.79,1.09)	0.93(079,1.09)

Abbreviations: BMI: body mass index; RWG: rapid weight gain; Early AR: early adiposity rebound.

Sensitivity analysis 1: adjusted for maternal age, family monthly income per capita, maternal education level, paternal education level, maternal pre-pregnancy BMI, paternal BMI, parity, maternal metabolic dysfunctions during pregnancy, alcohol use during pregnancy, tobacco use during pregnancy, iron supplementation during pregnancy, folic acid supplementation during pregnancy, children's sex, and exclusive breastfeeding duration ≥6 months.

Sensitivity analysis 2: adjusted for maternal age, family monthly income per capita, maternal education level, paternal education level, maternal pre-pregnancy BMI, paternal BMI, parity, maternal metabolic dysfunctions during pregnancy, alcohol use during pregnancy, tobacco use during pregnancy, iron supplementation during pregnancy, folic acid supplementation during pregnancy, children's sex, main caregivers before 3 years, screen time, and outdoor activity time.

*: $P < 0.05$; **: $P < 0.01$.

Table S8 Sensitivity analysis on the association between the trajectories of physical growth and early AR in children.

Physical growth indicators		Early AR[RR (95%CI)]	
		Sensitivity analysis 1	Sensitivity analysis 2
**Waist circumference**			
30-72 months			
	Normal trajectory	1.00	1.00
	High trajectory	2.52(2.19,2.91)**	2.51(2.18,2.89)**
	Very high trajectory	4.19(3.45,5.09)**	4.15(3.42,5.04)**
**Body fat**			
48-72 months			
	Normal trajectory	1.00	1.00
	High trajectory	3.02(2.63,3.48)**	3.00(2.61,3.45)**
**BMI**			
Birth-72 months			
	Low trajectory	0.78(0.64,0.95)*	0.78(0.64,0.96)*
	Rising trajectory	3.16(2.67,3.73)**	3.14(2.66,3.72)**
	Medium trajectory	1.00	1.00

High trajectory	1.18(0.99,1.41)	1.17(0.99,1.40)
Very high trajectory	2.86(2.35,3.48)**	2.81(2.31,3.41)**

Abbreviations: early AR, early adiposity rebound; BMI, body mass index.

Sensitivity analysis 1: adjusted for maternal age, family monthly income per capita, maternal education level, paternal education level, maternal pre-pregnancy BMI, paternal BMI, parity, maternal metabolic dysfunctions during pregnancy, alcohol use during pregnancy, tobacco use during pregnancy, iron supplementation during pregnancy, folic acid supplementation during pregnancy, children's sex, and exclusive breastfeeding duration ≥6 months.

Sensitivity analysis 2: adjusted for maternal age, family monthly income per capita, maternal education level, paternal education level, maternal pre-pregnancy BMI, paternal BMI, parity, maternal metabolic dysfunctions during pregnancy, alcohol use during pregnancy, tobacco use during pregnancy, iron supplementation during pregnancy, folic acid supplementation during pregnancy, children's sex, main caregivers before 3 years, screen time, and outdoor activity time.

*: P <0.05; **: P <0.01.

Table S9 Associations of BMI trajectories (using raw BMI data) and early AR in children.

Physical growth indicators		Crude model	Adjusted model
**Range of ages**	**Classification**	**Early AR [RR (95%CI)]**	**AR [RR (95%CI)]**
**BMI trajectories**			
Birth-24 months			
	Low BMI trajectory	1.15(0.99,1.45)	1.17(1.01,1.37)*
	Medium BMI trajectory	1.00	1.00
	High BMI trajectory	0.94(0.81,1.10)	0.90(0.78,1.05)
	Very high BMI trajectory	1.10(0.87,1.42)	0.99(0.77,1.27)
Birth-72 months			
	Low BMI trajectory	0.93(0.79,1.10)	0.94(0.80,1.11)
	Rising BMI trajectory	2.36(2.85,3.95)**	3.18(2.70,3.75)**
	Medium BMI trajectory	1.00	1.00
	High BMI trajectory	1.73(1.44,2.09)**	1.69(1.40,2.04)**
	Very high BMI trajectory	3.37(2.61,4.36)**	3.04(2.32,3.96)**

Model 1: Crude model.

Model 2: Adjusted for maternal age, family monthly income per capita, maternal education level, paternal education level, maternal pre-pregnancy BMI, paternal BMI, parity,

maternal metabolic dysfunctions during pregnancy, alcohol use during pregnancy, tobacco use during pregnancy, iron supplementation during pregnancy, folic acid supplementation during pregnancy, and children's sex.

*: P <0.05; **: P <0.01.

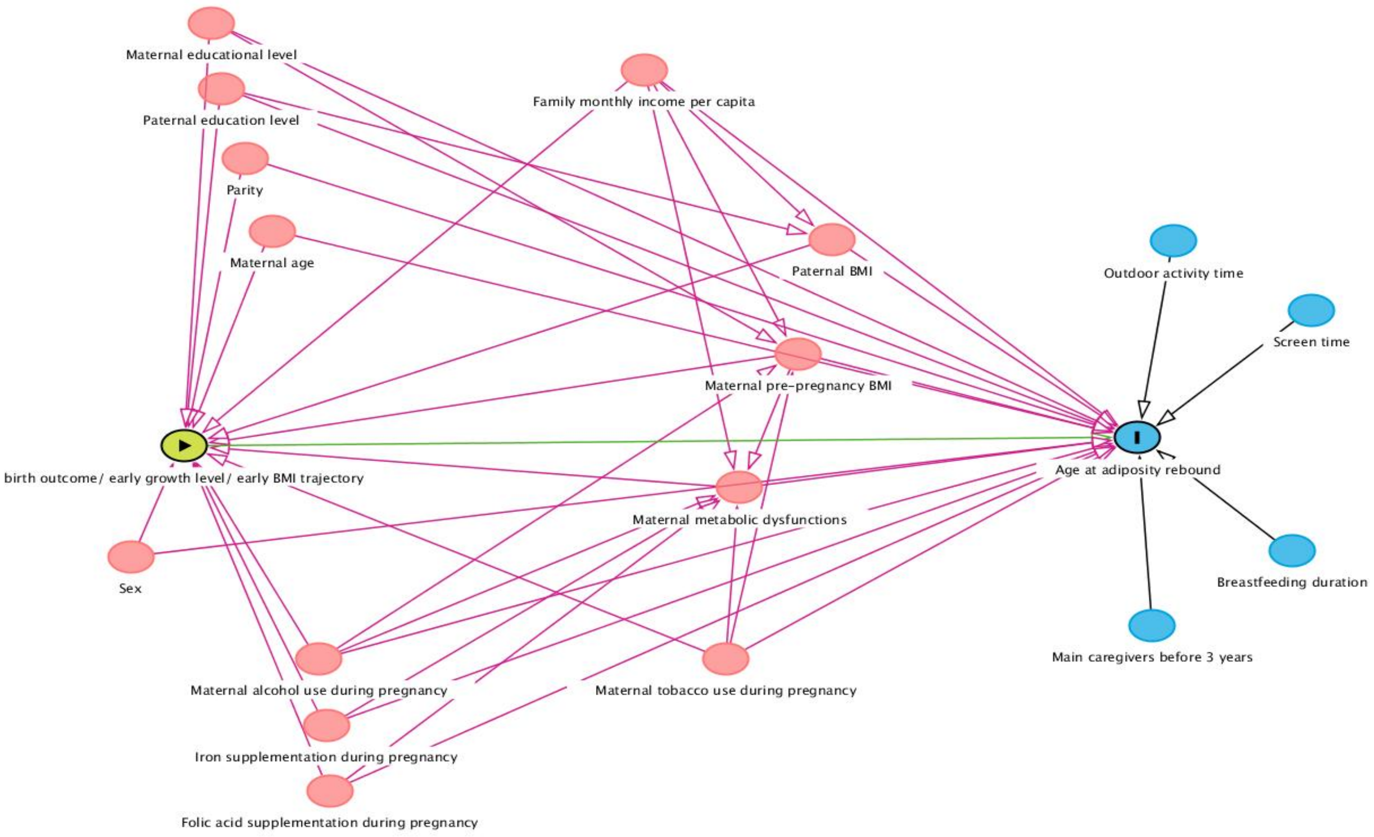

Figure S1 Directed acyclic graph of the relationship between birth outcome/early growth patterns and age at AR.

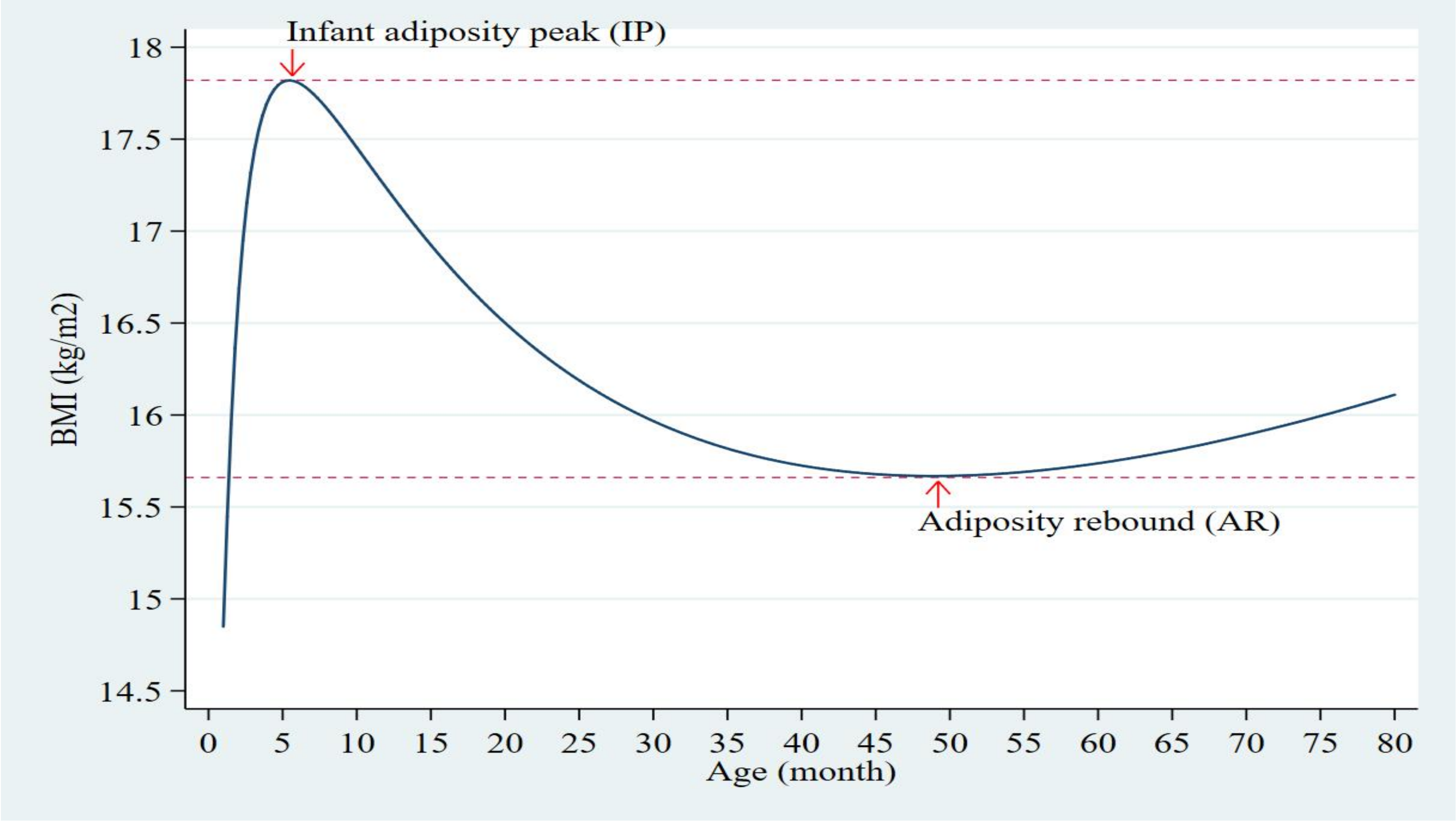

Figure S2 The overall trajectory plot based on the BMI fit of 2705 children.

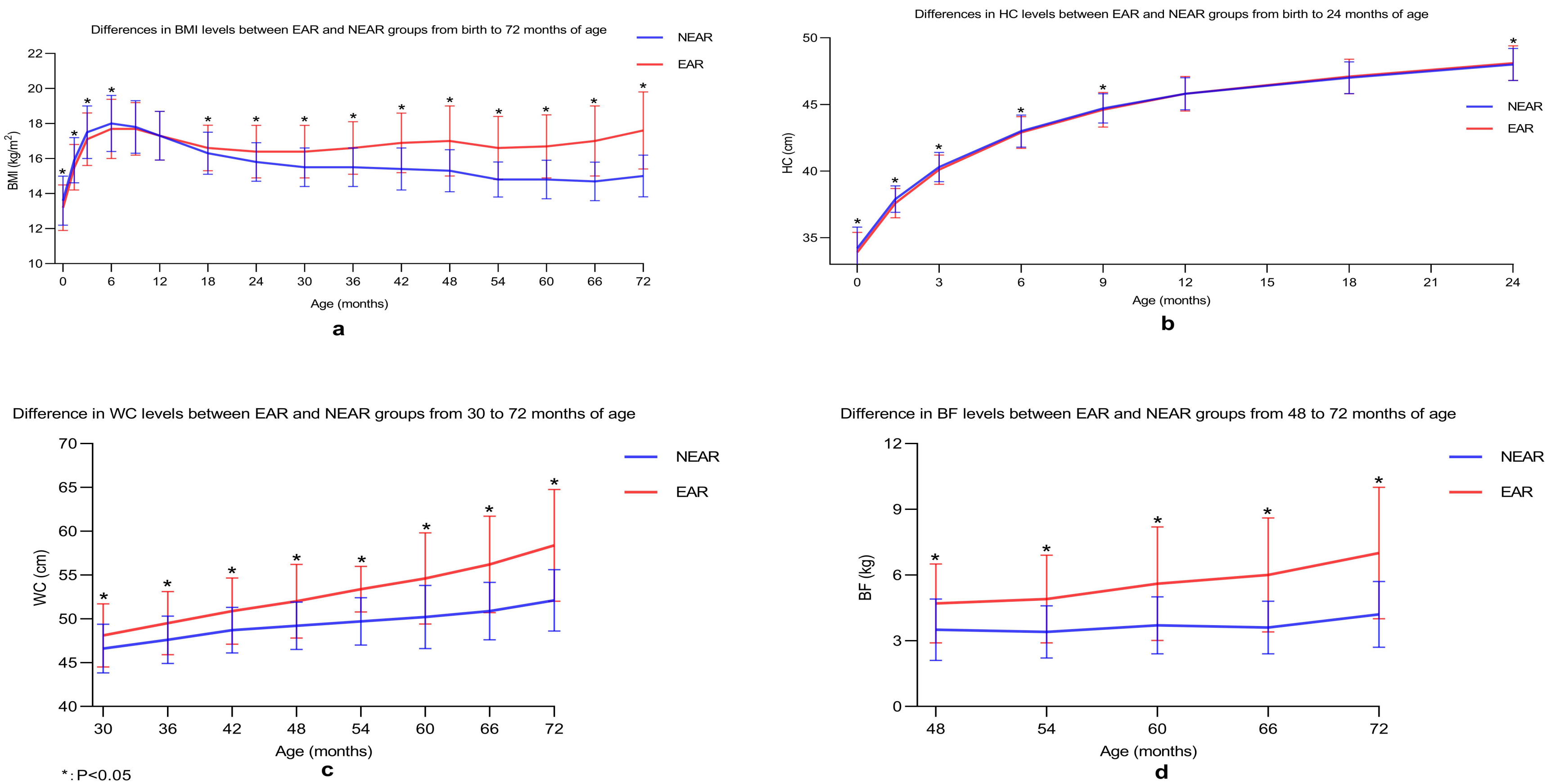

*: P<0.05

Abbreviations: EAR, early age at adiposity rebound; NEAR, non- early age at adiposity rebound; BMI, body mass index; HC. head circumference; WC, waist circumference; BF, body fat.

Figure S3 Physical growth trajectory of children in the early AR (EAR) and non-early AR (NEAR) groups.

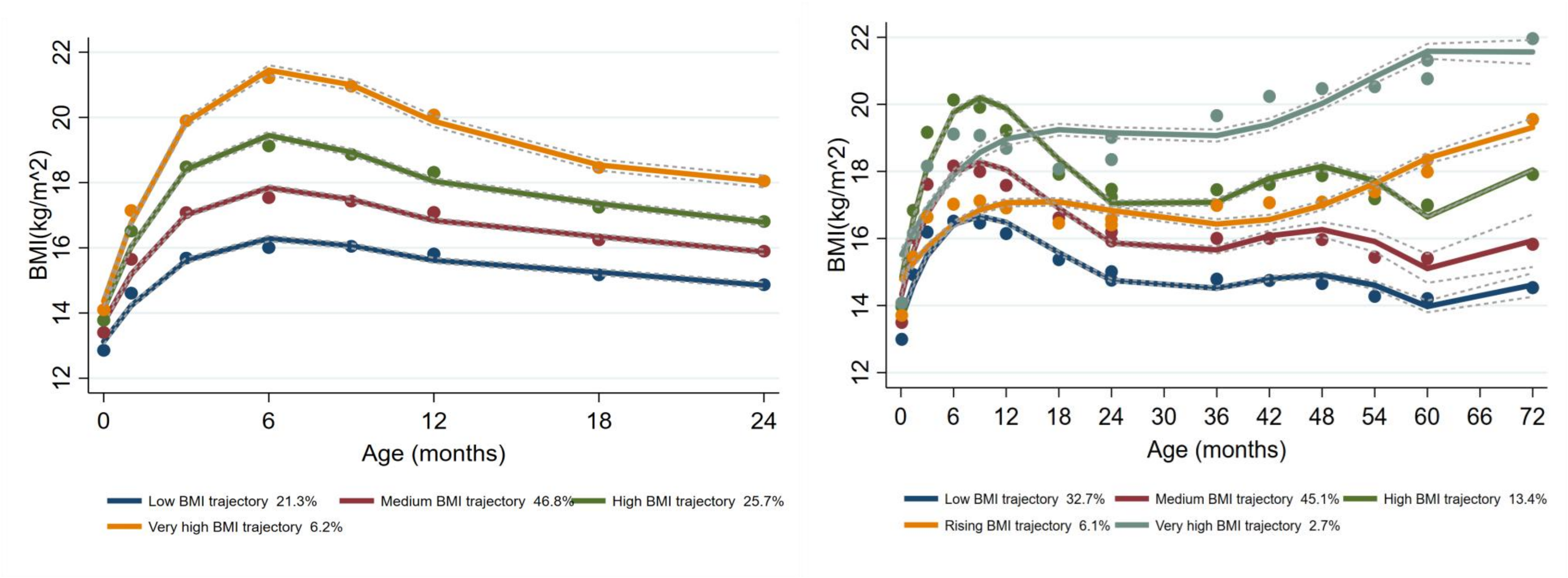

Figure S4 Trajectories fitted using raw BMI data.
